# Consecutive Lactation, Infant Birth Weight and Sex Do Not Associate with Milk Production and Infant Milk Intake in Breastfeeding Women

**DOI:** 10.3390/nu17061062

**Published:** 2025-03-18

**Authors:** Ashleigh H. Warden, Vanessa S. Sakalidis, Jacki L. McEachran, Ching Tat Lai, Sharon L. Perrella, Donna T. Geddes, Zoya Gridneva

**Affiliations:** 1 School of Molecular Sciences, The University of Western Australia, Crawley, WA 6009, Australia; ashleigh.warden@uwa.edu.au (A.H.W.); jacki.mceachran@uwa.edu.au (J.L.M.); ching-tat.lai@uwa.edu.au (C.T.L.); sharon.perrella@uwa.edu.au (S.L.P.); donna.geddes@uwa.edu.au (D.T.G.); 2 ABREAST Network, Perth, WA 6000, Australia; 3 UWA Centre for Human Lactation Research and Translation, Crawley, WA 6009, Australia; 4 Menzies School of Health Research, Royal Darwin Hospital Campus, Casuarina, NT 0810, Australia; vanessa.sakalidis@menzies.edu.au

**Keywords:** early nutrition, human milk, breastfeeding, consecutive lactation, milk production, milk intake, infant sex, birth weight, commercial milk formula

## Abstract

Background/Objectives: Optimal infant growth is reliant on the sufficient intake of human milk. Studies in animal models speculate that multiparous mothers produce a higher milk yield compared to primiparous mothers. We aimed to examine if there are relationships between consecutive lactations and infant demographics and both maternal 24 h milk production (MP) and infant milk intake (MI). Methods: Lactating mothers 1–6 months postpartum (*n* = 36; 25 fully breastfeeding (FBF), 11 supplementing with commercial milk formula (partly breastfeeding (PBF)) test-weighed their infants for 24 h during two consecutive lactations and provided demographic information. Twenty-four-hour MP by breast, infant MI (including mothers’ own expressed milk and formula), breastfeeding and expressing frequencies were measured. The statistical analysis used linear mixed modelling accounting for infant birth weight (FBF) or time postpartum (PBF) and for the random effect of the participant. Results: In the FBF group, there were no differences between lactations in terms of MP (*p* = 0.31) or the infant mother’s own MI (*p* = 0.14). The birth weight was higher for consecutive lactation infants (*p* = 0.008). Infant sex was not associated with MP (*p* = 0.12) or the infant mother’s own MI (*p* = 0.090). In the PBF group, the breastfeeding frequency (*p* = 0.042), MP (*p* = 0.025) and infant mother’s own MI (*p* = 0.019) were higher in consecutive lactations whilst formula intake was lower (*p* = 0.004). Conclusions: This study suggests that in fully breastfeeding women, there is no significant effect of consecutive lactation or infant sex on MP or infant MI during established lactation.

## 1. Introduction

Exclusive breastfeeding for the first 6 months of an infant’s life sets them up for healthy growth and development. Breastfeeding provides multiple nutrients, helps prevent many non-communicable diseases and confers numerous maternal health benefits [1,2]. A perceived low milk supply (LMS) negatively influences breastfeeding duration and self-efficacy [3] and is one of the major reasons reported for ceasing exclusive breastfeeding [4,5]. Australian and international reported rates of perceived LMS are 35–50% [3,5,6,7], yet studies of perceived LMS rarely measure actual infant milk intake to evaluate maternal perceptions. A recent study of predominantly pumping mothers showed that 84% of participants that reported LMS as a current breastfeeding difficulty indeed had low milk production (MP) [8], determined as <600 mL/24 h [9,10]. Whilst improving breastfeeding self-efficacy is vital in reducing mothers’ perception of LMS, understanding which factors place mothers at risk of actual LMS remains important [11,12].

Perceived LMS is more frequently reported by primiparous women (women that have been pregnant and given birth once [13]) [6] and it is thought that higher milk volumes are produced in consecutive lactations, yet there is a lack of human studies to confirm this. At 3–4 days postpartum, the perception of breast engorgement was more prevalent in multiparous women; however, no difference in MP was observed despite multiparas having a higher breastfeeding frequency than primiparas [14]. At one week postpartum, multiparous women were shown to produce significantly more breast milk (~140 mL) than primiparous women; however, this difference was lost by 4 weeks postpartum [15]. When a subset of these primiparous women measured 24 h MP during their second lactation, an increased MP (~125 mL) with the second infant was also found only at one week postpartum, with no significant difference at 4 weeks [16]. Further, one Australian study reported no association between parity and MP measured at 3.5 ± 1.4 months (min–max: 1–6 months) postpartum [10].

Several animal studies reported that multiparas produce a higher milk yield than primiparas. Primiparous Holstein cows had a lower 24 h MP than second- and third-parity cows, in which the MP increased and then decreased in subsequent lactations [17]. In rhesus macaques, parity was also significantly associated with milk yield [18]. Whilst not often comparable in methodology, animal milk yields are measured in those that solely provide their own milk to offspring, whilst in humans, supplementation with commercial milk formula may impact the capacity for full MP, thereby confounding the measurement of true lactation potential [12,19].

Several human and animal MP studies have also shown a potential offspring sex bias. During established lactation, Australian mothers of male infants produced an ~76 mL higher volume of milk than mothers of female infants [10]. Similarly, a UK study found that women produced 81 mL more milk for male infants [20] and a meta-analysis from studies conducted in 12 countries estimated that 50 mL more was produced for males [21]. Additionally, Iberian red deer mothers with male offspring had a higher milk yield [22], yet in rhesus macaques mothers of males, especially primiparas, produced lower milk yields [23,24]. Having female offspring was also related to a higher MP compared to having male offspring according to two large studies of Holstein [25,26] and Jerseys cows [26], though in the second study, accounting for lactation length differences due to longer gestation of male calves eroded most of the significance. Unfortunately, in animals, MP is not routinely measured with a validated reference method, making it impossible to compare studies. Furthermore, these relationships could be confounded by the infant birth weight which is higher in higher order infants [16] and may be positively associated with milk intake [27].

The aims of this small investigative study were to establish whether there is a difference in 24 h MP between two consecutive established lactations in fully breastfeeding (FBF) women as well as in those that partly breastfeed (PBF) and supplement with commercial milk formula. Additionally, we aimed to investigate associations between infant sex and birth weight and 24 h MP and infant milk intake.

## 2. Materials and Methods

### 2.1. Participants and Study Design

This retrospective longitudinal study is a secondary analysis of previously collected 24 h MP data and includes mothers (*n* = 36) during two consecutive lactations (L1 and L2) who provided breast milk to their infants aged between 2 weeks and 6 months and participated in various studies within our group. Mothers were recruited from the West Australian community through social media and recorded their MP between December 2011 and November 2024. The eligibility criteria were mothers of singletons with a birth weight ≥ 2500 g and birth gestation ≥ 37 weeks who were breastfed and/or consumed expressed breast milk (EBM). The exclusion criteria were mothers with a history of breast surgery which could affect MP.

All mothers provided their and their infants’ demographic information and written informed consent to participate in the study. The study was approved by The University of Western Australia Human Research Ethics Committee (2019/RA/4/20/6134) and conducted in accordance with the Declaration of Helsinki.

### 2.2. Measurements of 24-Hour Milk Production and Infant Milk Intake

Twenty-four-hour MP and infant milk intake were measured by the mothers at home at between 2 weeks and 6 months postpartum as infant intake of breast milk during this time is considered stable [10,28]. The mothers weighed their infant pre- and post-breastfeeding and supplementary feeds [10]. This was performed using a Medela Electronic Baby Weigh Scale (±2.0 g; Medela Inc., McHenry, IL, USA) for all feeds/expressions in one 24 h period plus one breastfeed/expression. The frequency of milk removal (number of milk removal sessions) was reported as the sum of breastfeeds and expression sessions over a 24 h period. Infant intake of their mother’s own milk was recorded as all breast milk consumed by breastfeeding and/or supplementary EBM feeds. Infant total milk intake was calculated as all breast milk consumed by breastfeeding and supplementary feeds of EBM, and commercial milk formula. Infant milk intake was calculated with the potential underestimate of 3–10% due to insensible water loss [29,30].

Twenty-four-hour MP reflects the milk synthesis rate and was calculated using the formula below, where *v_i_* is the volume of each breastfeed/expression, *N* is the total number of breastfeeds and expressions, and *T* is the elapsed time from the end of the first breastfeed/expression until the end of the last breastfeed/expression.

$$MP=\sum_{i=2}^{N}v_{i}\frac{24}{T}$$

Data were excluded if less than 20 h of time has elapsed from the end of the first breastfeed/expression as the synthesis rate may be reduced when the breast reaches its storage capacity [10]. All measurements of MP and milk intake were recorded and reported in grams as 1 g of milk is near equivalent to that of 1 mL (density: 1.03 g/mL) [31]. Milk production < 600 g/24 h was considered a low milk supply [9,10].

### 2.3. Anthropometric Measurements

The maternal height and weight at the time of 24 h MP, as well as the postpartum bra size, were reported by the mothers as a part of the demographics questionnaire. The body mass index (BMI) was calculated as kg/m^2^. The breast volume (cm^3^) of one breast was calculated based on both the bra cup size and band size, with reference to an online chart [32].

The infant naked weight was measured by the mothers at the start of the 24 h MP measurements. The infant weight-for-age (WAZ) z-scores at the time of 24 h MP measurements were determined using the World Health Organization (WHO) Infant Weight for Age Percentiles Clinical Calculator [33].

### 2.4. Statistical Analysis

In this longitudinal pilot study, the descriptive statistics are reported as the mean ± standard deviation (SD) and min–max or *n* (%) and modelling results are reported as the parameter estimate ± standard error (SE).

The analysis was conducted separately on the FBF (*n* = 25) and PBF (*n* = 11 at L1) subgroups with some of investigations (i.e., infant sex or birth weight relationships with 24 h MP parameters) restricted to the FBF group only, as mothers that feed commercial milk formula may not have reached their full lactation potential [12,19]. Twenty-four-hour MP was measured during two consecutive lactations. The power calculation was conducted using the ‘F tests–Linear multiple regression: Fixed model: R^2^ increase’ option in G*Power [34]. In the FBF group, 25 participants and two time points gave the study a power of 0.80 to detect an effect size of <0.16 with α = 0.05.

The comparison analysis of the groups’ means used paired Students *t*-tests to compare continuous variables, such breastfeeding frequency and volumes during 24 h MP, and Chi-square or Fisher’s exact tests for the categorical variables such as parity, infant sex and birth mode between the FBF and PBF groups.

Relationships between 24 h MP parameters across lactations, and between 24 h MP parameters and maternal and infant demographics were assessed using univariable and multivariable linear mixed effect models with the participant as a random effect (FBF and PBF groups) and infant birth weight (FBF group) or time postpartum (PBF group) as a confounder. Due to the small sample size of PBF group and potential confounding of the feeding mode (and further size reduction in case of accounting for sex or birth weight), no results for the infant birth weight and sex relationships with 24 h MP parameters are presented.

The statistical analysis was performed in R 4.4.2. The significance level was set at <0.05. Missing data were dealt with using available case analysis.

## 3. Results

### 3.1. Participant Characteristics

Thirty-eight participants satisfied the selection criteria but two were excluded due to having data restricted to less than 20 h of time elapsed from the end of the first breastfeed/expression. The two lactations were on average 2.3 ± 0.9 years apart (min–max: 1.0–5.0 years). At L2, the mothers in the PBF group had a higher BMI compared with the FBF mothers (*p* = 0.005) and infants in the FBF group had a higher birth weight (*p* = 0.001) (Table 1).

### 3.2. Infant Birth Weight

In the univariable modelling in the FBF group, the infant birth weight was higher at consecutive lactation (235.86 (62.70) g, *p* = 0.001) and lower in infants of mothers with a higher BMI (−29.05 (12.58) g, *p* = 0.031) but did not differ by infant sex (male: 11.01 (93.85) g, *p* = 0.91). The infant birth weight was positively associated with the total milk intake (0.14 (0.07) g, *p* = 0.048) and average breastfeed volume (0.02 (0.01) g, *p* = 0.017).

### 3.3. Consecutive Lactations

**Figure 1:**
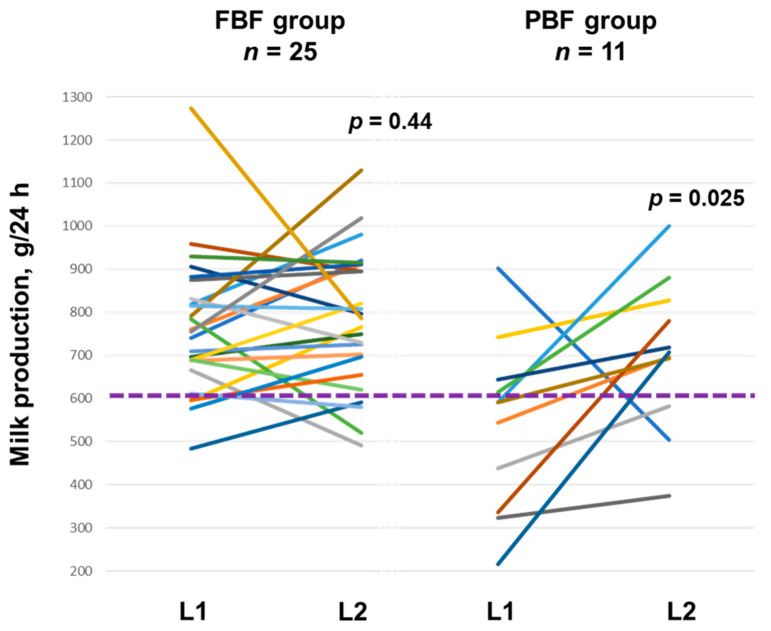
Milk production during lactation 1 (L1) and lactation 2 (L2) in fully breastfeeding (FBF) and partly breastfeeding (PBF) women. Each line represents an individual participant. *P*-values are from linear mixed effect models accounting for infant birth weight (FBF) or time postpartum (PBF). Purple dotted line divides participants with a normal vs. low milk supply [9,10].

The 24 h MP parameters at L1 and L2 are presented in Table 2 and Figure 1. In the FBF group, there was no systematic difference between L1 and L2 in 24 MP and infant milk intake parameters in the univariable models or after accounting for the infant birth weight (Table 3).

**Figure 2:**
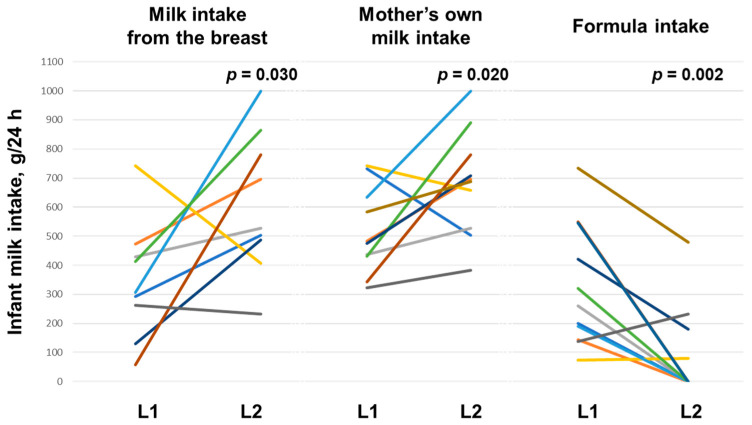
Infant milk intake during lactation 1 (L1) and lactation 2 (L2) in partly breastfeeding (PBF) women. Each line represents an individual participant. *p*-values are from linear mixed effect models accounting for time postpartum.

In the PBF group, in univariable modelling the infants’ intake of mothers’ own milk (96.41 (27.92) g, *p* = 0.006) and 24 h MP (91.68 (24.60) g, *p* = 0.004) were positively associated with time (month) postpartum. The infant milk intake from the breast (*p* = 0.030) and intake of mother’s own milk (*p* = 0.020) were higher during L2, whilst the intake of commercial formula milk was lower (*p* = 0.002) (Table 4). After accounting for time postpartum, these relationships persisted, and 24 h MP (*p* = 0.025) and breastfeeding frequency (*p* = 0.042) became significantly higher during L2 (Table 4, Figure 2).

When compared to the FBF group, at L1, the mothers from the PBF group had a lower 24 h MP (*p* < 0.001), breastfeeding frequency (*p* = 0.038), average breastfeed volume (*p* = 0.038), milk intake from the breast (*p* < 0.001), and mother’s own milk intake (*p* < 0.001); however, the expressing frequency (*p* < 0.001) and infant EBM intake (*p* = 0.015) were higher in the PBF group (Table 2). At L2, the expressing frequency (*p* = 0.033) and infant intake of expressed breast milk (*p* = 0.007) remained higher in the PBF group, whilst the average breastfeed volume remained lower (*p* = 0.038), with no difference in all other 24 h MP and milk intake parameters (Table 2).

### 3.4. Infant Sex

In the FBF group in the univariable modelling, infant sex was not associated with 24 h MP or any other 24 h MP parameter except for the expressing frequency, which was higher in mothers of male infants (*p* = 0.049); this relationship persisted after accounting for birth weight (*p* = 0.046, Table 5).

### 3.5. Relationships Between 24 h Milk Production Parameters and Maternal Factors

In the univariable modelling in both the FBF and PBF groups, a higher milk removal frequency was associated with a lower average breastfeed volume (FBF: −4.12 (0.54) g, *p* < 0.001; PBF: −3.48 (0.93) g, *p* = 0.006); this relationship persisted after accounting for birth weight or time postpartum (FBF: −3.86 (0.44) g, *p* < 0.001; PBF: (−3.25 (0.89) g, *p* = 0.008; respectively). Milk removal frequency was not associated with 24 h MP in both the FBF and PBF groups in the univariable models (FBF: −8.88 (4.77) g, *p* = 0.075; PBF: −5.0 (9.60) g, *p* = 0.61) or after accounting for birth weight or time postpartum (FBF: −8.24 (4.30) g, *p* = 0.069; PBF: −2.74 (7.62) g, *p* = 0.73).

In the univariable modelling in FBF group, older mothers had a higher expression frequency (0.22 (0.09), *p* = 0.022); this relationship persisted after accounting for birth weight (0.24 (0.09), *p* = 0.018). The infant total milk intake was higher in infants of mothers with a larger bra cup volume (0.06 (0.08) g, *p* = 0.042) only after accounting for birth weight.

In the univariable modelling in the PBF group, the breastfeeding frequency was higher in mothers with a higher BMI (0.41 (0.17), *p* = 0.048); however, after accounting for time postpartum, the significance was lost (0.43 (0.18), *p* = 0.055) but the maternal bra cup volume became positively associated with 24 h MP (0.17 (0.07) g, *p* = 0.042).

## 4. Discussion

This pilot longitudinal study focused on breastfeeding mothers that measured their MP during two consecutive lactations and found no significant increase in MP or changes in any 24 h MP parameters amongst the FBF mothers. In the women that supplemented with commercial milk formula (PBF) during L1, there was a significant increase in 24 h MP (131 (49) g) and infant breast milk intake (181.22 (63.69) g), which may be partially due to an increase in breastfeeding frequency (3.86 (1.56)); however, the total number of milk removal sessions was not different by lactation. Additionally, infant sex was not associated with 24 h MP or milk intake.

### 4.1. Consecutive Lactations

This study failed to find a significant difference in MP between two consecutive lactations in contrast to animal studies that report higher milk yields in multiparous mothers compared to primiparous mothers [17,18]. The difference may lie in the methodology in animal models that often employ anaesthesia, such as in Iberian red deer and rhesus macaques [22,23,24,35], which may impact the single ’yield’ sample collected that is also used to estimate 24 h MP. In both ruminants and primates, milk ejection is required for the effective removal of milk. In animals that possess cisterns such as deer, goats and cows [36], the cisternal milk can be removed without triggering milk ejection, resulting in a significant underestimation of MP [37]. Further, the individual impact of sedation is unknown and various factors may suppress oxytocin release such as stress and sudden stimuli (i.e., changing calves or switching between a calf and machine milking) [38]. In humans, very little milk can be removed in the absence of milk ejection [39,40].

Human studies of consecutive lactations are rare; however, perceived LMS is reportedly more common in primiparous women [6]. Some studies have reported higher MP volumes for consecutive lactations in the first few days postpartum. One study showed that Japanese multiparous women hand-expressed higher volumes of colostrum in the first 30 h postpartum compared to primiparous women [41], which is likely related to experience or familiarity with the hand-expressing technique. Another study showed the milk intake of 4-day-old infants of Italian multiparous women was greater than the infants of primiparous women [42]. Two UK studies have also shown lower 24 h MP in primiparous women compared to multiparous women [15] and a significant increase in MP in these primiparous women during their second lactation when measured at 7 days postpartum [16]. This difference disappeared when measured at 4 weeks in established lactation. Consistent with these finding, other studies have shown no association of parity with infant milk intake in well-nourished populations [10,43,44,45]. Only infants of Gambian mothers with 10 or more children displayed lower milk intakes [46], but this level of parity is not common and may be confounded by maternal age [47]. It is therefore likely that increased volumes during the first week postpartum are due to more robust secretary activation in multiparous women. Delayed secretory activation reported amongst primiparous women [48] has been proposed to be due to primiparous females having less secretory tissue, which results in less milk being synthesized and stored in the breast compared to multiparous females.

In grey seals, primiparous females had a smaller average alveolar size and a higher cell density throughout lactation compared with multiparous females, indicating that their secretory cells are smaller and less-developed [49]. As a decrease in cell density is consistent with an increase in cell development and size as lactation progresses, this may partly account for the increase in 24 h milk yield amongst grey seals. The alveolar density also was higher at the start of lactation in primiparous females, but at peak lactation, there was no difference between the groups, potentially translating to no difference in milk yield during established lactation. Indeed, murine models showed that stable loss of DNA methylation in a Stat5a-biased fashion during the first pregnancy resulted in the mammary glands from parous animals responding more robustly to a subsequent pregnancy [50], with glands of multiparous mice displaying an earlier response to pregnancy hormones with a greater number of ductal structures and an earlier detection of milk protein expression in cells. However, on day 12, the mammary glands from both parous and nulliparous female mice produced similar amounts of milk proteins [50], again suggesting that established lactation may not be affected, similar to the results in our and other human studies.

The human mammary glands may indeed respond faster in consecutive lactation, which might be, in part, a result of having an earlier first breastfeed (within 1 h of birth) and using less formula in the hospital [51] or feeding more frequently in the days after birth [14]. Interestingly, whilst increased milk removal frequency is associated with greater MP and infant milk intake during the early postpartum period (days/weeks) [19,52,53,54] as well as during established lactation [55], the milk removal frequency did not change from one established lactation to another in the FBF group, consistent with the lack of change in MP. However, in the PBF group, despite the small increase in breastfeeding frequency, the combined frequency of breastfeeding and breast expression (milk removal frequency) did not differ by lactation and was not associated with 24 h MP. This suggests that the increased MP in this group might indeed be due to either priming of molecular pathways such as Stat5a described above or a post-feed upregulation of miRNA species that may contribute to cell division activation in order to facilitate the generation of more milk-secretory lactocytes [56]. The total milk removal frequency in this group did not increase significantly as the number of breastfeeds may have increased at the expense of breast expressions, though the latter not significantly differ between L1 and L2. Using a breast pump to remove additional milk after breastfeeds has been shown to increase MP by 15–40% [44,57]. Additionally, the infant only ‘empties’ the breast once a day and may remove less available milk during a breastfeed (on average 67%) [10] compared to electric pumps, which have shown to remove up to 75% of breast milk [58,59,60].

There was, however, a positive relationship between 24 h MP and time postpartum in the PBF group, indicating that in this population, some improvements are seen within a lactation as mothers apply the acquired knowledge and skills and increase the breastfeeding frequency. Whilst the PBF group increased breast milk feeding, only 73% of the mothers produced a full MP (≥600 g, Figure 1). Therefore, infant feeding intentions, as well as non-modifiable causes of reduced MP, such as pregnancy complications, obesity and genetics, should be considered as frequent milk removal may not resolve the effects of aberrant development of the breast in puberty and pregnancy (proliferation and differentiation) [52,61,62]. These findings should be verified in a larger cohort.

Maternal age has been associated with lower MP in successive lactations in Holstein cows where the MP decreased in the fourth and the subsequent lactations [17]. Whilst small human studies have found no associations between maternal age and infant milk intake [44,63], maternal parity ≥ 10 has been associated with lower infant milk intake [46] and a recent large study (*n* = 609) showed an average decrease of 8 mL/24 h in MP with every one-year increase in maternal age or one-unit increase in BMI [47]. In our study, there were no differences in the FBF group with increasing age and this is likely due to the relatively low participant numbers. The increased breast milk intake of infants in the PBF group suggests molecular changes may be more potent than increasing maternal age and could be a target for potential therapies for women with LMS [64].

### 4.2. Infant Birth Weight

Our study showed that in the FBF group, the infant birth weight was higher at consecutive lactation, which has been reported previously for higher birth orders [16,65,66], with significant factors being a longer gestational age, an increased number of prenatal care visits during the pregnancy, and the sex of the infants. Animal studies have attributed a smaller adult body mass in firstborns to a lower milk yield and transfer of fewer calories and essential nutrients, as in rhesus macaques [35]. However, this relationship could be reversed, with infants potentially feeding according to their birth weight and growth demands. In our study, in the FBF group, the infants with higher birth weight had higher average breastfeed volume and breast milk intake. There is a gap in the literature when it comes to healthy term infants, with only one report of infants with a higher birth weight consuming more milk during the first 14 days of life [27]. Additionally, those that were encouraged to feed more frequently initially took more milk (725 vs. 502 mL/24 h), but this difference was not confirmed at 1 month of age, despite continuation of frequent nursing [52]. Further work is needed to clarify whether MP in mothers is controlled solely by the infant’s appetite/growth demands during the period of established lactation.

### 4.3. Infant Sex

In our study, infant sex was not associated with 24 h MP or any other infant-related 24 h MP parameters, which contrasts with several animal studies that have indicated a potential sex bias for MP. Drawing systematic conclusions from these studies, however, is challenging as most have limitations in methodology. In two large bovine studies, having female offspring was associated with greater MP compared to having male offspring [25,26], though the first study used standardized lactation curves to predict monthly data points and in the second study, adjusting for the shorter lactation length for male calves removed most of the significance. Having high MP in dairy cows is generally associated with reduced fertility, health and survival [25], so these data are not representative of the norm for the species. In rhesus macaques, the mothers of male offspring produced lower milk yields [23,24], but in both of these studies, the MP was estimated from a single milking session with the animals sedated. In contrast, Iberian red deer had higher milk yields with male offspring, but again, MP was estimated from a single milking session under sedation and after an isolation period from the offspring [22].

In humans, during established lactation and using reference methods for determining MP, the mothers of male infants reportedly produced a 50–81 mL higher volume of milk than mothers of females in 14 studies from countries across five continents [10,20,21]. However, as some of these studies were conducted using the isotope tracer method, non-milk water intake could have confounded the results. Alternatively, our study may be underpowered for this type of analysis since we did not account for insensible water loss [29,30]. The sex-biased milk synthesis could be in part a representation of the birth weight relationship with infant milk intake, as male infants are usually heavier than females, and this was not significantly different in our study. Furthermore, the mothers of male infants expressed milk more frequently than the mothers of females (Table 5); however, the total milk removal was not different.

The strength of this pilot study includes the measurement of 24 h MP and infant milk intake using a validated reference method [67]. The main limitation of this study is the modest sample size, although it is the largest study of this kind to date. We did not account for maternal diet or hydration status, although the effect of additional fluids consumed by breastfeeding mothers on MP remains unknown due to a lack of well-designed studies [68]. Further, our sample is relatively homogenous (predominantly Caucasian and healthy breastfed singletons from urban mothers of higher social-economic status) where malnutrition is unlikely; thus, our results may not be transferable to more diverse populations. Additionally, our sampling method may be biased as the participants that approach our research group with breastfeeding challenges may have sought research participation to address these challenges or they are supportive of and dedicated to breastfeeding, thus participating again after the birth of another child.

## 5. Conclusions

This study suggests that in fully breastfeeding women, there is no effect of consecutive lactation or infant sex on 24 h milk production or infant milk intake during established lactation. However, women that supplement with commercial milk formula may indeed have a higher milk production in a subsequent lactation, provided that there are no intrinsic causes for the low milk supply, suggesting that early interventions such as breastfeeding education and support could address low milk supply issues in some women.

## Figures and Tables

**Table 1:** Participant characteristics.

Maternal and Infant Characteristics	FBF L1 (*n* = 25)	FBF L2 (*n* = 25)	*p*-Value ^2^	PBF L1 (*n* = 11)	PBF L2 (*n* = 11)	*p*-Value ^3^	*p*-Value ^4^	*p*-Value ^5^
Maternal age (years)	31.4 ± 3.3 ^1^	33.6 ± 3.4	**<0.001**	33.1 ± 3.3	35.3 ± 3.3	**<0.001**	0.15	0.19
	(25–39)	(27–41)		(29–40)	(31–42)			
Parity (Multiparous)	4 (16.0)	25 (100.0)	**<0.001**	3 (27.3)	11 (100.0)	**0.001**	0.65	1.00
	(1–3)	(2–4)		(1–3)	(2–4)			
Maternal body mass index (kg/m^2^)	25.1 ± 4.2 ^6^	25.3 ± 4.0	0.40	28.6 ± 5.5 ^7^	30.2 ± 5.5	0.44	0.063	**0.005**
	(19.5–36.9)	(18.5–36.5)		(20.3–37.1)	(19.4–37.7)			
Maternal breast volume (cm^3^)	746 ± 300 ^6^	782 ± 288 ^8^	0.51	939 ± 569 ^7^	939 ± 387 ^7^	1.00	0.22	0.22
	(390–1180)	(310–1580)		(480–2340)	(480–1810)			
24 h MP time (months postpartum)	3.1 ± 1.3	2.8 ± 1.0	0.27	2.4 ± 1.5	2.6 ± 1.3	0.71	0.15	0.77
	(1.0–6.3)	(0.7–4.5)		(0.6–4.9)	(1.4–6.0)			
Race	*n* = 24			*n* = 11				
Caucasian	22 (91.6)			10 (90.9)				
Asian	1 (4.2)			1 (9.1)				
Other	1 (4.2)			NA				
Infant sex (Male)	10 (40.0)	11 (44.0)	0.78	7 (70.0)	7 (70.0)	1.00	0.29	0.47
Infant birth gestation (weeks)	39.2 ± 1.0 ^6^	39.3 ± 0.7	0.55	39.2 ± 0.9 ^9^	39.2 ± 1.1	0.97	0.95	0.78
	(37–41)	(38.1–41)		(38–41)	(37.4–41.3)			
Infant birth weight (g)	3259 ± 353 ^6^	3494 ± 269	**0.001**	3436 ± 280 ^9^	3547 ± 397	0.59	0.17	0.64
	(2666–4050)	(2800–3985)		(2915–3810)	(2930–4170)			
Infant weight at 24 h MP (g)	5454 ± 1362	5556 ± 999	0.78	4742 ± 1355	5275 ± 1316	0.25	0.16	0.49
	(3540–8872)	(3964–7306)		(2958–7015)	(3638–8445)			
Infant WAZ z-score at 24 h MP	−0.50 ± 1.47	0.11 ± 1.04	0.059	−0.72 ± 1.50	−0.36 ± 0.94	0.41	0.68	0.21
	(−3.9–2.99)	(−1.67–1.63)		(−3.42–1.62)	(−2.02–0.73)			
Birth mode	*n* = 22	*n* = 23		*n* = 10	*n* = 10			
Vaginal	11 (50.0)	14 (60.9)	0.46	3 (30.0)	6 (60.0)	0.37	0.45	1.00
Elective caesarean	6 (27.3)	7 (30.4)	1.00	4 (40.0)	4 (40.0)	1.00	0.68	0.70
Non-elective caesarean	5 (22.7)	2 (8.7)	0.24	3 (30.0)	0 (0.0)	0.21	0.68	1.00

^1^ Data are shown as the mean ± standard deviation and min–max or *n* (%). ^2^ *p*-value indicates the difference between the FBF L1 and FBF L2 participants; ^3^ *p*-value indicates the difference between the PBF L1 and PBF L2 participants using Chi-square and Fisher’s exact tests or paired Student’s *t*-tests where appropriate. ^4^ *p*-value indicates the difference between the FBF L1 and PBF L1 participants. ^5^ *p*-value indicates the difference between the FBF L2 and PBF L2 participants using unpaired Student’s *t*-tests. Bold font indicates a significant difference. FBF, fully breastfeeding; L1, lactation 1; L2, lactation 2; MP, milk production; NA, not available or not applicable; PBF, partly breastfeeding (and supplementing with commercial milk formula); WAZ, weight-for-age. ^6^ *n* = 23; ^7^ *n* = 9; ^8^ *n* = 24; ^9^ *n* = 10.

**Table 2:** Milk production parameters and breastfeeding characteristics.

Parameter	FBF L1 (*n* = 25)	FBF L2 (*n* = 25)	PBF L1 (*n* = 11)	PBF L2 (*n* = 11)	*p*-Value ^2^	*p*-Value ^3^
24 h milk production (g)	739 ± 125 ^1^	761 ± 157	511 ± 198	661 ± 171	**<0.001**	0.096
	(536–1111)	(501–1065)	(219–927)	(406–967)		
Breast milk expressed (g)	137 ± 137 ^4^	161 ± 156 ^5^	244 ± 193 ^6^	323 ± 289 ^7^	0.15	0.19
	(12–469)	(15–500)	(8–610)	(16–708)		
Breastfeeding frequency	12.7 ± 4.0	11.6 ± 3.1	9.5 ± 3.8	13.3 ± 4.2	**0.038**	0.21
	(6–23)	(6–22)	(4–15)	(8–20)		
Expressing frequency	1.2 ± 1.9	1.2 ± 2.7	6.0 ± 4.4	3.9 ± 4.5	**<0.001**	**0.033**
	(0–8)	(0–12)	(0–14)	(0–14)		
Milk removal frequency	14.0 ± 4.4	12.9 ± 3.7	14.6 ± 3.8	14.8 ± 5.3	0.66	0.22
	(7–26)	(6–22)	(9–22)	(7–24)		
Average breastfeed volume (g)	60 ± 25	65 ± 20	40 ± 25 ^6^	49 ± 22 ^8^	**0.038**	**0.049**
	(30–128)	(30–113)	(6–98)	(23–78)		
Milk intake from the breast (g)	699 ± 170	733 ± 203	350 ± 191 ^6^	611 ± 242 ^8^	**<0.001**	0.15
	(395–1156)	(230–1130)	(57–742)	(232–1000)		
Expressed breast milk intake (g)	37 ± 103	26 ± 60	153 ± 169 ^6^	172 ± 244	**0.015**	**0.007**
	(0–420)	(0–212)	(0–440)	(0–689)		
Infant intake of mothers’ own milk (g)	736 ± 144	759 ± 193	519 ± 149 ^6^	672 ± 178	**<0.001**	0.21
	(472–1156)	(230–1130)	(323–742)	(382–1000)		
Commercial milk formula intake (g)	0 ± 0	0 ± 0	325 ± 211	89 ± 154	NA	NA
	NA	NA	(73–734)	(0–480)		
Total milk intake (g)	736 ± 144	770 ± 185	797 ± 229	761 ± 211	0.34	0.89
	(472–1156)	(230–1130)	(462–1317)	(504–1169)		

^1^ Data are shown as the mean ± standard deviation. ^2^ *p*-value indicates the difference between the FBF1 and PBF1 participants. ^3^ *p*-value indicates the difference between the FBF2 and PBF2 participants using unpaired Student’s *t*-tests. Bold font indicates a significant difference. FBF, fully breastfeeding; L1, lactation 1; L2, lactation 2; NA, not applicable; PBF, partly breastfeeding (and supplementing with commercial milk formula). ^4^ *n* = 12; ^5^ *n* = 8; ^6^ *n* = 10; ^7^ *n* = 7; ^8^ *n* = 9.

**Table 3:** Systematic differences between two consecutive lactations in the fully breastfeeding (FBF) group (*n* = 25).

Parameter	Univariable Model *n* = 50 ^3^			Multivariable Model *n* = 48 ^3^			
	PE	(SE)	Predictor *p*-Value	PE	(SE)	Predictor *p*-Value	Birth Weight *p*-Value
24 h milk production (g)	21.79 ^1^	(30.72)	0.49	24.66 ^2^	(30.70)	0.43	0.31
Breast milk expressed (g)	−14.24	(32.59)	0.67	7.32	(34.91)	0.84	0.47
Breastfeeding frequency	−1.08	(0.90)	0.24	−1.29	(1.01)	0.22	0.59
Expressing frequency	0.00	(0.65)	1.00	0.07	(0.74)	0.93	0.86
Milk removal frequency	−1.08	(0.91)	0.25	−1.26	(1.08)	0.26	0.71
Average breastfeed volume (g)	4.61 ^4^	(4.91)	0.36	2.96 ^5^	(5.22)	0.58	0.054
Intake of mothers’ own milk (g)	23.04	(41.24)	0.58	11.85	(44.24)	0.79	0.14
Milk intake by the breast (g)	33.88	(47.22)	0.48	11.15	(50.59)	0.83	0.14
Expressed breast milk intake (g)	−10.84	(22.79)	0.64	−0.15	(23.45)	1.00	0.97
Total milk intake (g)	34.64	(40.51)	0.40	22.44	(43.17)	0.61	0.11

^1^ Data are shown as the parameter estimate ± standard error of measurement (PE ± SE) from univariable mixed effect modelling and ^2^ after accounting for infant birth weight. ^3^ Number of observations. ^4^ *n* = 49 observations; ^5^ *n* = 47.

**Table 4:** Systematic differences between two consecutive lactations in the group that was partly breastfeeding (PBF) and supplementing with formula (*n* = 11).

Parameter	Univariable Model *n* = 22 ^3^			Multivariable Model *n* = 22 ^3^			
	PE	(SE)	Predictor *p*-Value	PE	(SE)	Predictor *p*-Value	TP *p*-Value
24 h milk production (g)	149.72 ^1^	(68.93)	0.055	130.67 ^2^	(48.45)	**0.025**	**0.003**
Breast milk expressed (g)	−15.82	(102.49)	0.88	−13.39	(105.28)	0.90	0.78
Breastfeeding frequency	3.71 ^4^	(1.64)	0.054	3.86 ^4^	(1.56)	**0.042**	0.36
Expressing frequency	−2.09	(1.76)	0.26	−1.87	(1.76)	0.32	0.18
Milk removal frequency	0.18	(1.97)	0.93	0.21	(2.01)	0.92	0.85
Average breastfeed volume (g)	8.66 ^4^	(10.78)	0.45	6.60 ^4^	(10.38)	0.55	0.15
Intake of mothers’ own milk (g)	200.91	(72.89)	**0.020**	181.22	(63.69)	**0.019**	**0.005**
Milk intake from the breast (g)	261.33 ^4^	(99.57)	**0.030**	242.15 ^4^	(95.75)	**0.039**	0.15
Expressed breast milk intake (g)	18.91	(89.39)	0.84	21.34	(91.76)	0.82	0.75
Commercial milk formula intake	−236.91	(58.98)	**0.002**	−230.18	(59.79)	**0.004**	0.28
Total milk intake (g)	−36.00	(53.66)	0.52	−46.67	(49.16)	0.37	0.077

^1^ Data are shown as the parameter estimate ± standard error of measurement (PE ± SE) from univariable mixed effect modelling and ^2^ after accounting for time postpartum. ^3^ Number of observations. Bold font indicates a significant difference. TP, time postpartum. ^4^ *n* = 19 observations.

**Table 5:** Infant sex (male) relationships with 24 h milk production parameters in the fully breastfeeding (FBF) group (*n* = 50).

Parameter	Univariable Model *n* = 50 ^3^			Multivariable Model *n* = 48 ^3^			
	PE	(SE)	Predictor *p*-Value	PE	(SE)	Predictor *p*-Value	Birth Weight *p*-Value
24 h milk production (g)	36.38 ^1^	(37.53)	0.34	10.91 ^2^	(34.73)	0.76	0.12
Breast milk expressed (g)	44.64	(32.44)	0.18	51.75	(32.11)	0.12	0.48
Breastfeeding frequency	−1.88	(1.00)	0.072	−1.95	(1.00)	0.065	0.25
Expressing frequency	1.31	(0.63)	**0.049**	1.41	(0.66)	**0.046**	0.89
Milk removal frequency	−0.76	(1.11)	0.50	−0.68	(1.16)	0.56	0.35
Intake of mothers’ own milk (g)	−3.66	(47.74)	0.94	−30.29	(45.53)	0.51	0.080
Milk intake from the breast (g)	−16.16	(53.20)	0.76	−54.68	(49.10)	0.28	0.090
Expressed breast milk intake (g)	12.22	(24.06)	0.62	25.49	(21.85)	0.26	0.98
Total milk intake (g)	12.84	(46.74)	0.79	−13.42	(44.31)	0.77	**0.0499**

^1^ Data are shown as the parameter estimate ± standard error of measurement (SE) from univariable mixed effect modelling and ^2^ after accounting for infant birth weight. ^3^ Number of observations. Female sex is the reference. Bold font indicates a significant difference.

## Data Availability

Restrictions apply to the availability of some, or all data generated or analysed during this study due to ethical reasons. The corresponding author will on request detail the restrictions and any conditions under which access to some data may be provided.

## Abbreviations

The following abbreviations are used in this manuscript:

BMI	Body mass index
EBM	Expressed breast milk
FBF	Fully breastfeeding group
L1	Lactation 1
L2	Lactation 2
LMS	Low milk supply
MP	Milk production
PBF	Partly breastfeeding and supplementing with commercial milk formula group
TP	Time postpartum
